# Multi-population analysis of the Cuban SARS-CoV-2 epidemic transmission before and during the vaccination process

**DOI:** 10.1063/5.0066912

**Published:** 2021-10-08

**Authors:** D. Guinovart-Sanjuán, R. Guinovart-Díaz, K. Vajravelu, W. Morales-Lezca, I. Abelló-Ugalde

**Affiliations:** 1 Department of Mathematics, University of Central Florida, 4393 Andromeda Loop N, Orlando, Florida 32816, USA; 2 Departamento de Matematicas, Universidad de La Habana, San Lazaro y L 10400, La Habana, Cuba; 3 Universidad de La Habana, Centro de Estudios para el Perfeccionamiento de la Educación Superior (CEPES). La Habana, Cuba

## Abstract

In this work, several mathematical models for the spread of viruses and diseases are presented. In particular, the work focuses on the coronavirus disease 2019 (COVID-19) pandemic. A multi-population model is presented for the study of the interaction of various populations and the contagion of the virus between them. A second model on vaccination is presented, which allows analyzing the behavior of the disease taking into account the effectiveness of the vaccine and the speed of COVID-19 after the vaccination process. Finally, both models are applied to analyze the epidemic in Cuba. For this study, the official data reported by the Cuban Ministry of Health from March 2020 to August 2021 is used.

## I. INTRODUCTION

The COVID-19 pandemic has become a huge event for the human species that has affected more than 167 millions individuals around the world. In a broad sense, the virus depends on the movement of its host to spread.^1^ The spreading is different and it varies according to the population, the location, the local interventions; like the use of masks and social distancing;^2,3,31^ as well as the important vaccination of the population.^4,5^

At this time, several mathematical models allow describing the behavior of the pandemic under different situations.^6–10^ In Ref. 11, a generalized SEIR model is presented. That model allows subdividing the population into seven different groups. These groups are: susceptible, exposed, infected, quarantined, recovered, deceased, and protected. This model has proven to be efficient and has been used to model the behavior of the pandemic in several countries.^12–14^

However, we have focused our research to study how the spread of COVID-19 varies according to populations and their vaccination. For this, we have developed two mathematical models based on the generalized SEIR model presented in Ref. 11. The first model provides multi-population analysis, which describes the interaction among different populations and the interaction within the same population.^15,16^ The second model takes into account a single population but adds to the protection parameter a vaccination factor that includes the speed of vaccination of the population and its efficacy.^17,18^

These models were applied to study the spread of the virus on the island of Cuba. For this, the official data of active, deceased, and recovered cases reported by the Ministry of Public Health of Cuba were taken from 19. In the first model, the population of Cuba is divided between the capital with a population of more than 2.1 million inhabitants in an area of less than 730 km^2^ and the rest of the island with a population of 9.2 million inhabitants in a region of more than 109 000 km^2^. The behavior of both subdivisions of Cuba was analyzed during the first year of the pandemic. In the second model, the inclusion of the vaccination process was taken into account. For this, the data of the vaccine candidates presented by the Ministry of Public Health (MINSAP)^20^ were considered. An analysis was carried out of the different scenarios that could occur if we take into account different vaccination speeds and how the effect of vaccination can protect the population. Finally, it is studied how the spread of the epidemic is accelerated in the different provinces of Cuba. It is determined for which provinces said acceleration may indicate a decrease or an increase in daily cases.

## II. GENERALIZED SEIR MATHEMATICAL MODELS

### A. Multipopulational model of generalized SEIR model

**FIG. 1.**
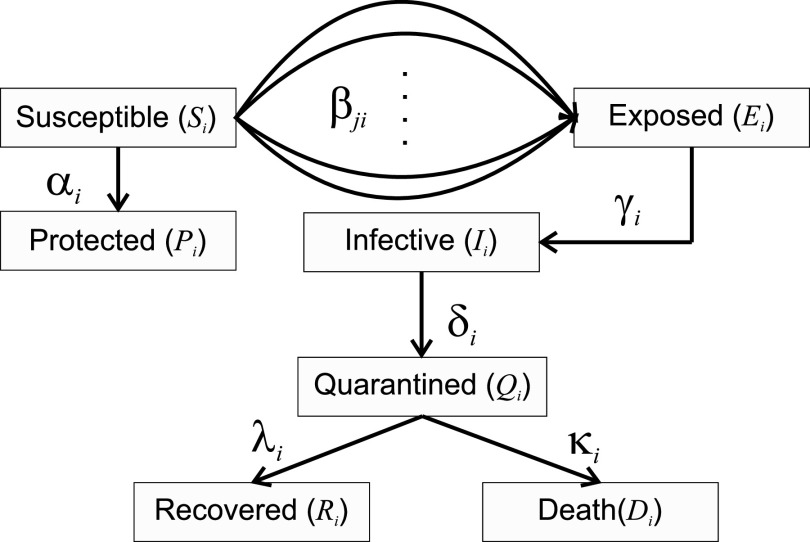
Flow diagram of the generalized SEIR model with multiple populations.

To describe the pandemic of the COVID-19 and the interaction of several populations, a generalization of the model presented in^11^ is used. In that study, *m* populations are taken into account. However, our model considers that the member of the susceptible group of the population *i*, *S_i_*, can be infected by a group of infected people of the population *j*, *I_j_*, with a rate of *β_ij_*. Each population has an *α_i_* parameter that denotes the protection rate of each population and it depends on the local interventions. This parameter allows the group members of the susceptible (*S_i_*) to move to the protected group (*P_i_*). The members of the populations that have been exposed to the virus (*E_i_*) by their interaction with an infected one of any of the populations become infested with a probability of *γ_i_*. The $\gamma_{i}^{-1}$ parameter is the average latency period of the disease. In some cases, it is necessary to hospitalize or isolate certain infested. The *δ_i_* parameters determine the probability that an infected person passes into the quarantined group (*Q_i_*). Another group of people who cannot overcome the severity of the symptoms of the disease die (*D_i_*) with a *λ_i_* mortality rate and those who recovered to do so with a *κ_i_* rate. The flow diagram of the generalized SEIR model with multiple populations is illustrated in Fig. 1.

Therefore, the dynamical system of equations that describes the interaction among the populations is given by the system

$$\begin{array}{c} \frac{dS_{i}}{dt}=-\sum_{j=1}^{m}\beta_{ij}\frac{S_{i}I_{j}}{N}-\alpha_{i}S_{i},\\ \frac{dE_{i}}{dt}=\sum_{j=1}^{m}\beta_{ij}\frac{S_{i}I_{j}}{N}-\gamma_{i}E_{i},\\ \frac{dI_{i}}{dt}=\gamma_{i}E_{i}-\delta_{i}I_{i},\\ \frac{dQ_{i}}{dt}=\delta_{i}I_{i}-\lambda_{i}Q_{i}-\kappa_{i}Q_{i},\\ \frac{dR_{i}}{dt}=\lambda_{i}Q_{i},\\ \frac{dD_{i}}{dt}=\kappa_{i}Q_{i},\\ \frac{dP_{i}}{dt}=\alpha_{i}S_{i}, \end{array}$$ (1)

where *S_i_*, *P_i_*, *E_i_*, *I_i_*, *Q_i_*, *R_i_*, *D_i_* represent the number of susceptible, protected, exposed, infected, quarantined, recovered, and death, respectively, for the population *i*. A constant population *N_i_* is considered (non-demographic) for each *i*, therefore $N_{i}=S_{i}+P_{i}+E_{i}+I_{i}+Q_{i}+R_{i}+D_{i}$ and $N=\sum N_{i}$. Finally, the parameter *α_i_* represents the protection rate, *β_ij_* is the infection rate, $\gamma_{i}^{-1}$ is the average latent time, $\delta_{i}^{-1}$ is the average quarantine time, *λ_i_* is the cure rate and *κ_i_* is mortality rate of the corresponding population.

### B. Mathematical modeling of the vaccination process (SEIQRDPV)

The SEIQRDPV model defines the groups of a population that are threatened or affected due to the spread of COVID-19. This kind of model is standard and has been commonly used in the vaccination modeling of other virus-induced diseases^13^ among others.

Vaccines are rarely perfect and have an immune shelf life. For the analysis of the vaccination model, we have taken that the efficacy of the vaccine is given by the ratio *η*. This parameter describes the average percentage of effectiveness of the vaccines that are being applied to control the virus or disease. Due to the margin of failure of the vaccine, the model considers the possibility of infection of individuals who are in the protected compartment (*P*) with a rate β(1−η).

**FIG. 2.**
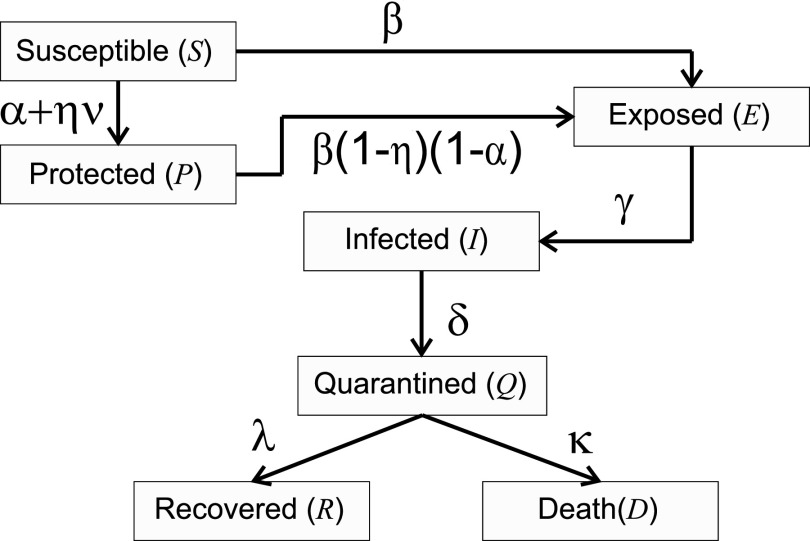
Flow chart for the vaccination model on population.

Another parameter that influences the protection of the population is the vaccination rate *ν*. The parameter *ν* takes values that depend on the resources, the population, and the availability. In the model described in Fig. 2, the vaccination process impacts a direct transfer of people from compartment (*S*) to compartment (*P*) due to the appearance of antibodies in the immune system of vaccinated people. To develop the vaccination process, a schedule is introduced where the vaccination is administered and the daily vaccination rate is estimated in each period. Each period is determined by a time interval $t_{k}\leq t\leq t_{k+1}$ where the difference $t_{k+1}-t_{k}$ denotes the length of the period in days. In each period, the vaccination rate is constant; therefore, ν(t) represents a step function, i.e., $\nu (t)=\nu_{k}$ if $t\in (t_{k},t_{k+1})$.

Finally, the dynamical system that described the vaccination period for one population is given by

$$\begin{array}{cc} & \frac{dS}{dt}=-\beta \frac{SI}{N}-\alpha S-\eta \nu S,\\ & \frac{dE}{dt}=\beta \frac{SI}{N}-\gamma E+\beta (1-\eta )(1-\alpha )\frac{PI}{N},\\ & \frac{dI}{dt}=\gamma E-\delta I,\\ & \frac{dQ}{dt}=\delta I-\lambda Q-\kappa Q,\\ & \frac{dR}{dt}=\lambda Q,\\ & \frac{dD}{dt}=\kappa Q,\\ & \frac{dP}{dt}=(\alpha +\eta \nu )S-\beta (1-\eta )(1-\alpha )\frac{PI}{N}. \end{array}$$ (2)

### C. Estimation of the parameters of the models

For the estimation of the parameters of the systems (1) and (2), the algorithm used in 11 was modified and adapted to derive the corresponding parameters. The code uses the MATLAB function *lsqcurvefit* and a fourth-order Runge–Kutta method. The code can be downloaded from Ref. 21. Here we can see that the estimation of the parameters is a least-square optimization process with a confidence region strategy.^30^ It is a Levenberg–Marquardt,^22–24^ algorithm whose initial approximations are generally very close to the optimal ones sought, so the process converges rapidly. Because the SARS-CoV-2 transmission rate varies significantly over time, in this work the estimation of the parameters was performed by subdividing the entire period into 15-day time intervals. It is a known fact,^25^ that almost any infection with a prevalence profile can be perfectly adapted to a SIR or SEIR model with a variable transmission rate, this fact is very close to the reported data used in the estimation.

Notice that both models include a parameter *α* (0<α<1). That measures the behavior of the population, i.e., if the population isolates itself, uses masks for protection, maintains distance, etc. The analysis of the variation of the parameters in the different time intervals can indicate to us when we have neglected or not the protection and its importance to achieving the control of the epidemic. In Ref. 26, these parameters *β* and *α* have been compared in different periods, allowing us to reach conclusions for the maintenance of the interventions taken by the governments.

## III. APPLICATIONS OF THE MATHEMATICAL MODELS FOR THE STUDY OF THE COVID-19 PANDEMIC IN CUBA

### A. Cuba data and multi-population prediction of daily positive cases (*Q_i_*) until February 2021

Cuba's data were retrieved from the official Health Department of Cuba database.^19,20^ The data obtained is classified by regions, age, and gender. During our investigation, we noticed that the pandemic behaved differently in Havana and the rest of the island, since Havana is the main tourist destination. For our study, we have divided Cuba into two main regions, i.e., two populations: one is the capital, Havana, and the rest of the island, denote as non-Havana region. On the other hand, the activity time of the pandemic has been divided into four periods. These periods were determined from the decision-making of the country's authorities and how the said decisions affected in one way or the other the behavior of the pandemic. The first period comprises from March 12th (the day when the first case was detected) to June 8th; where the country closed the schools, public transportation, and foreign tourism. A second period from June 9th to September 5. During this period, international tourism continue to be closed, but national tourism was open, and the movement of the population increases due to the summer season. The third period corresponding to the time from September 6 to December 3, due to the tough economic situation, the country partially reopens to European tourism. In addition, Cubans stranded abroad return and a large part of the Cuban diaspora visits their relatives on the island. PCR is carried out at airports on all visitors, but they stay in the houses of their families. Finally, the fourth period from December 4 to February 20 (the day where we close the data collection), this period is characterized by new year activities and an increase in visits from Cubans living abroad.

**FIG. 3.**
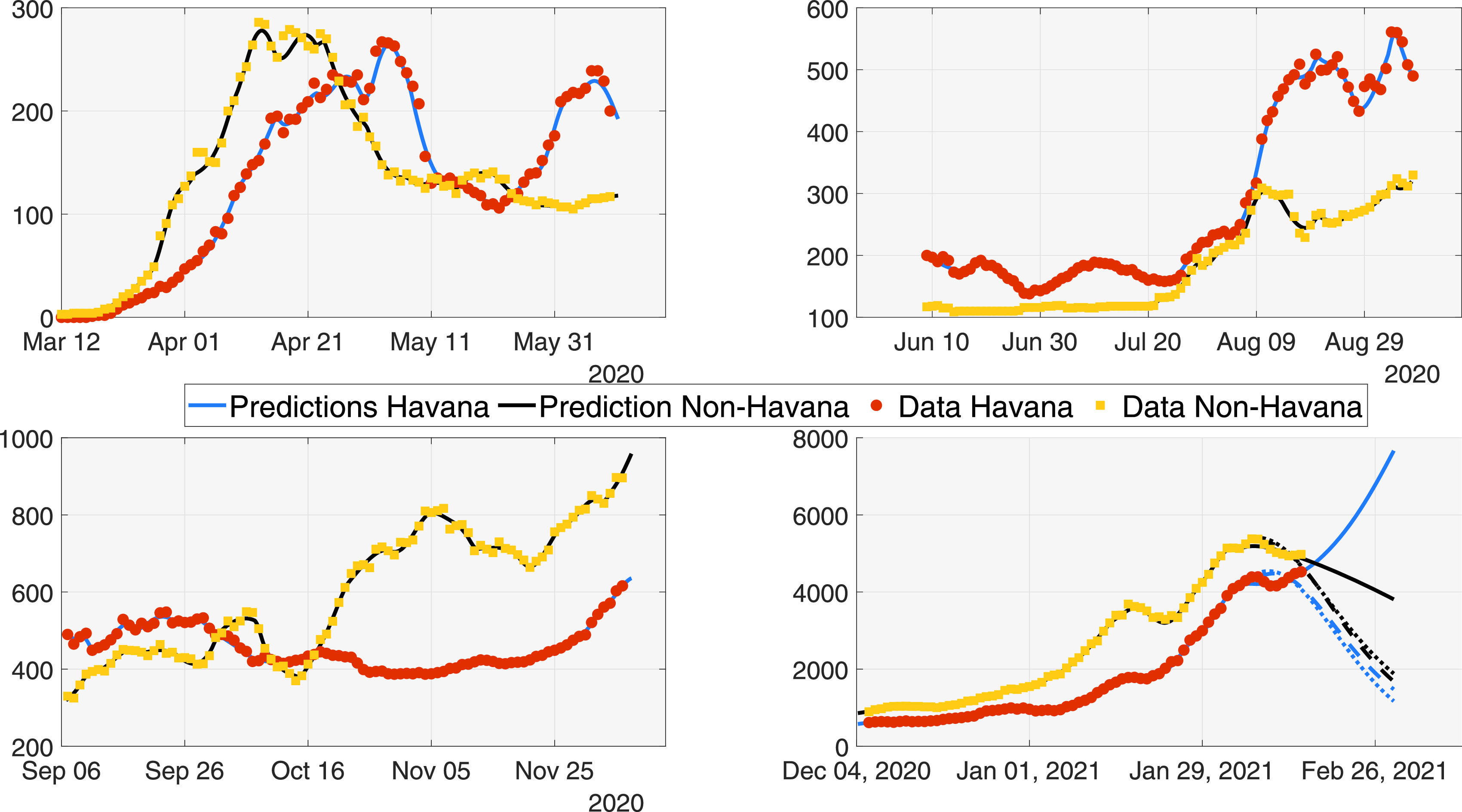
Prediction of the active cases using multi-population method to study the first year of the pandemic in Cuba. Period 1 (from March 12 to June 8); period 2 (from June 9 to September 5); period 3 (from September 6 to December 3); period 4 (from December 4 to February 20).

In Fig. 3, the data of the active cases from Havana and non-Havana regions during the four periods are illustrated. At the same time, the approximations obtained by our model over the data are shown. These approximations use the information from 21 days before the prediction. This allowed us to validate the accuracy of our model and at the same time analyze and draw conclusions about what happened in each of the periods.

During the first period, the non-Havana area began to have an increase in cases because the virus arrived in Cuba through an airport located in the central area of the island, far from the capital Havana. In these months, Cuba remains open to foreign tourism outside of Havana. From May to July, Cuba was closed to tourism. According to official data reported by the government, there was a period of control of the pandemic. During this time, isolation interventions, mandatory use of masks, and social distancing were imposed. At the same time, schools and recreation centers were closed. By mid-August, Cuba partially reopened its doors for tourism from Europe, which arrived through Havana. Despite the interventions, a new outbreak of the pandemic occurred in Havana, the most densely populated part of the island with a population density of almost 3000 inhabitants per square kilometer. In the following periods, Cuba remained open, which brought a gradual growth of the pandemic reaching almost 10 000 active cases. In the period from December to February (fourth period), the activities of the new year and the restart of the school year, consequently, brought an acceleration in the cases in these months. This may have led the country to make the decision to gradually decrease the influx of tourists to the island in early March 2021.

With the model described by the system (1), we have been able to recreate different scenarios. Taking into account the behavior of the pandemic with 7, 14, and 21 days in advance. Figure 3 shows the predictions made by our model for these cases, where an increase in cases could be noted in Havana. In February 2021, Cuba partially closed its entry of tourists according to the official media.

For this study, we have only focused on the location of the individuals and have set aside parameters that include age, gender, and other factors important to the behavior of the pandemic. All this is due to the lack of data or published studies that allow estimating the parameters.

### B. Cuba vaccination prediction

The model explained in the Sec. II B is applied to the estimation of the behavior of COVID-19 in Cuba, according to the vaccination strategy disclosed by the Cuban Ministry of Health (MINSAP).^20^ The MINSAP has established that the main vaccination period has been divided in seven months May to December. To do this, it has been defined the function *ν* with constant values in each month. The vaccination function *ν* is defined in seven intervals given by all the months between May and December. The number of people completely vaccinated during May was equivalent to 0.82%, in June it was the 11.1%, and in July 12%. The average vaccination constants of the mentioned months are $\nu_{1}=0.111/30$, $\nu_{2}=0.12/30$, and $\nu_{3}=0.115/30$, respectively. For the remaining months of the year 2021, it is estimated an increase in 15% of people will be vaccinated each month. With this schedule, the end of the year would be reached with 90% of the population immunized.

The vaccination period started on May 12 (*t*_0_) and the end day will be *t_n_*. Based on the data of confirmed, hospitalized, recovered, and deceased that are disclosed every day by the MINSAP in a daily report, we have made several simulations considering the vaccination model (2).

**FIG. 4.**
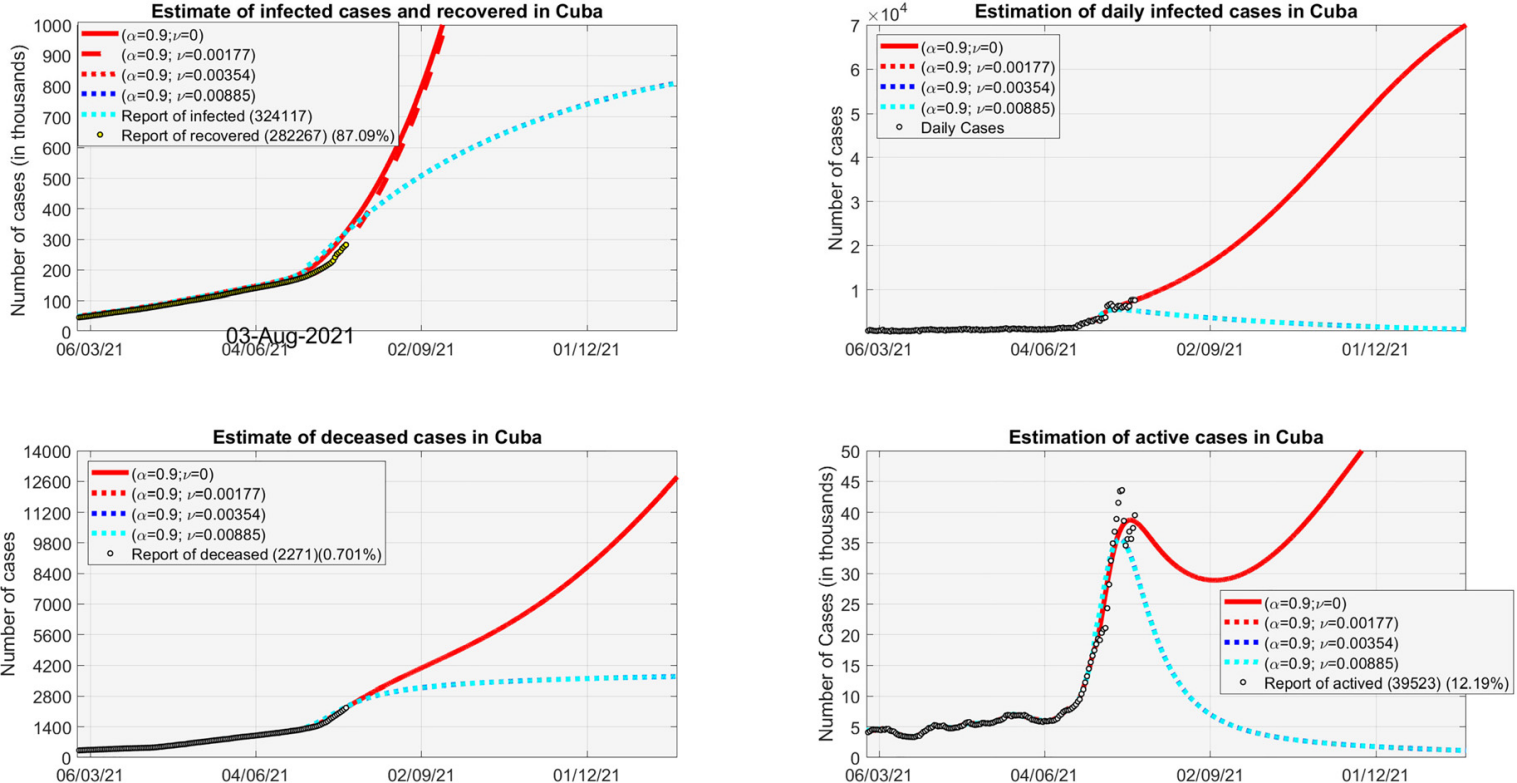
Estimate of the recovered, infected, deceased, and active cases in Cuba considering a protection rate of 0.9 and different vaccination speed rates.

Figure 4 shows how the different vaccination schedules influence the following parameters: immunized, daily confirmed, daily deceased, and active cases. The data were taken from May 15 and 21 days ago to estimate the parameters of the system (Sec. II B). The solid line red graph represents an estimate of what could occur in the absence of a vaccination schedule. Then different schemes are considered: slow, intermediate, average, fast. It can be concluded from the simulations presented in the figure that the most convenient thing is to have a rapid vaccination scheme that guarantees that the population is immunized as soon as possible. In all cases, there is a delay and the effects of immunization take time to be observed. We have considered an efficacy of 90% in the case of the vaccine candidates that are being used. Rapid vaccination guarantees much earlier to lower daily incidence and deaths.

With about 600 thousand immunized in the Havana (capital of Cuba, more than 2 million of inhabitants), the death toll could be lowered to below one per day on average as of November 2021. The daily confirmed cases would be below three by the end of the year; therefore, the hospital system would have already recovered its capacity to attend the rest of the services. Other restrictions could be removed and Havana would regain its economic capacity. However, in the absence of vaccines, it is necessary to increase the protection or isolation of people and reduce the concentration points of people. Figure 4 shows how the number of active cases can decrease when strong restrictive measures are taken. We know that these measures are not sustainable for long, but they must be prioritized when the transmission is very high and their application must be local.

### C. Phenomenological models to study the pandemic in Cuba

In the work 27, the authors applied phenomenological models to the study of COVID-19 in Cuba. Only the public daily report, made by the health authorities of the country since the beginning of the epidemic, is used as the data source. It is shown that phenomenological models have great forecasting value, and their prognoses can guide the interventions carried out by national health systems to contain the expansion of COVID-19.

**FIG. 5.**
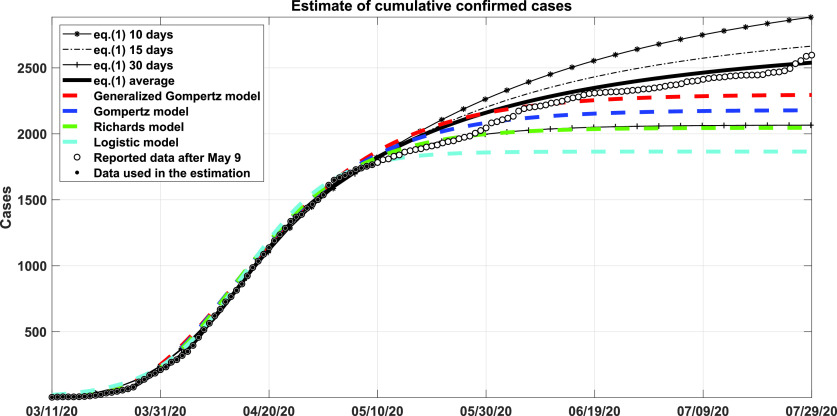
Comparison of model Eq. (1) with phenomenological models. The generalized Gompertz model and the average models maintain a good prediction of accumulated cases even after 30 days of the end of dates.

A comparison of the model (1) considering only the population of Cuba (single population) with the phenomenological models reported in 27 is shown in Fig. 5. The period corresponds from March 11 to July 29, 2020. In order to predict the cumulative confirmed cases, the parameters in (1) are estimated considering three different intervals: 10, 15, and 30 days. An average curve from the three estimations is also illustrated in Fig. 5. The results obtained from the generalized Gompertz model^27^ and the average models (1) provide a good prediction of accumulated cases, even after 30 days.

An advantage of the equation model (1) over the phenomenological models are shown in Fig. 3, as it provides the possibility of estimating the number of people hospitalized (quarantine), fundamental information for the Ministry of Health. The different predictions obtained with the model (1) considering various intervals for the estimations allow obtaining a cone of possible outcomes for the behaviors of the pandemic. Another advantage is the possibility to modify this type of model and study the impact of different conditions, like vaccination, as expressed in system (2).

## IV. ACCELERATION OF CONFIRMED CASES IN CUBA

**FIG. 6.**
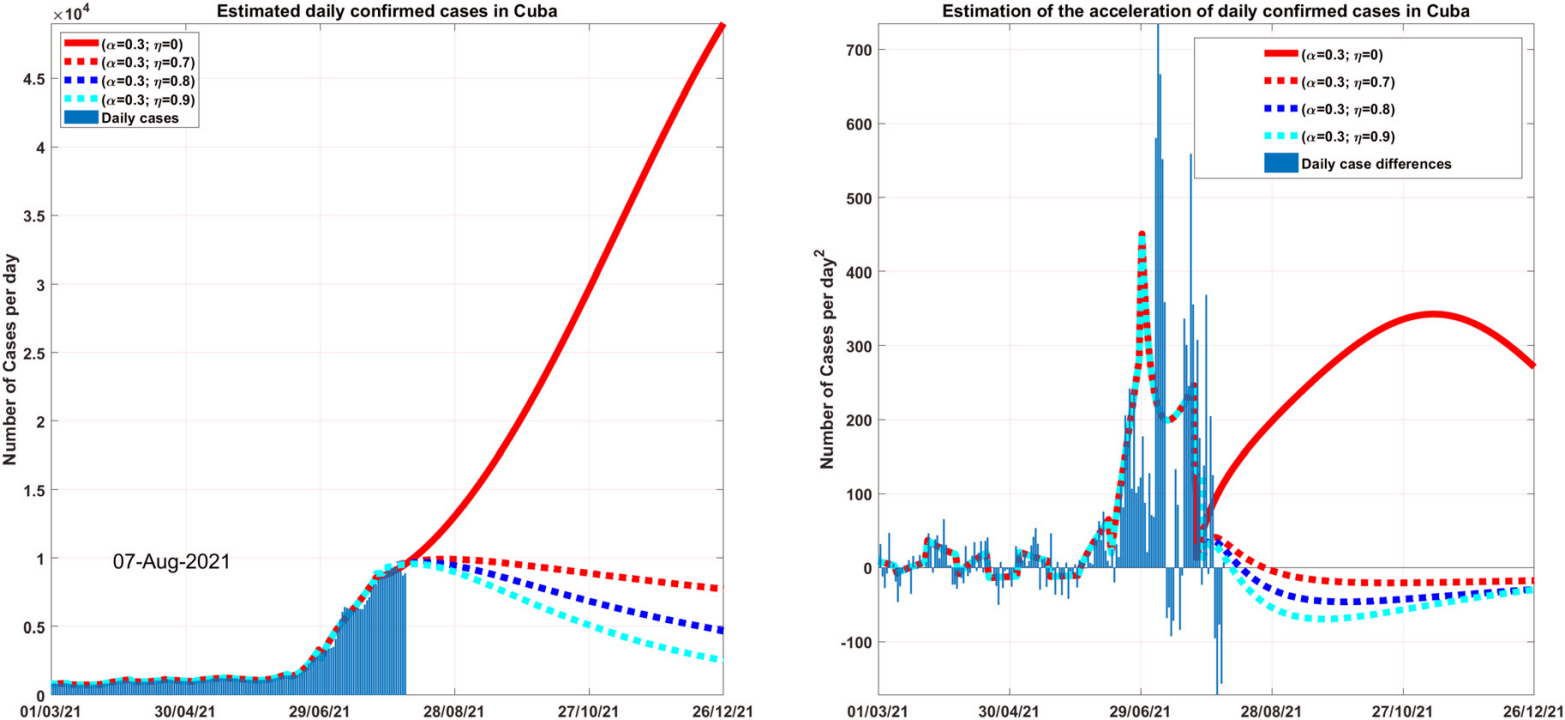
Comparison between the confirmed cases in Cuba (left) and the daily acceleration of the cases (right).

One of the ways that we have used the most to visualize COVID-19 data in Cuba has been tracking the daily behavior of new confirmed cases. In Fig. 6 (left), the numbers represented by the blue bars constitute the cases reported daily and the continuous red line is considered as the speed of the cases that are reported daily in Cuba from March 1, 2021, to August 6, 2021. The projection of this speed into the future if there is no vaccination indicates that growth may be unstoppable, reaching increases of more than 40 thousand cases at the end of October and about 60 thousand for year fines. If the vaccination process continues to reach a figure of 20 thousand vaccinated daily and for different values of the effectiveness of the vaccine against new strains, a very convenient reduction in the speed of confirmed cases could be achieved, reaching by the end of October values below 5000 confirmed cases and below 3500 by the end of the year.

Another way of studying the behavior of the epidemiological situation is presented in Fig. 6 (right) where the acceleration rate of confirmed cases is shown.^28^ To compute the acceleration, the following formula is used:

$$\frac{d^{2}}{dt^{2}}(Q+R+D)=\delta \gamma E-\delta^{2}I.$$ (3)

The deduction of this formula can be found in Appendix B. The number of new cases is accelerated or slowed down at this rate. Note that in case the vaccination process is not carried out (solid red line), the acceleration would increase from July 26, indicating a faster growth of confirmed cases. Looking at the data, this way is helpful because the rate at which cases are increasing is a reasonable indicator of how intense that wave might be and how long it might last. For example, if in Cuba the vaccination process continues at the rate of about 20 thousand vaccinated daily, the models indicate that the acceleration of confirmed cases may remain negative in the coming days and with a tendency to remain constant and close to zero. Showing that the daily incidence will have a decreasing trend, but it will do so slowly, if the same vaccination speed is maintained and the protection of the population is only 30%.

Under these conditions, 48 thousand cases would be avoided in next 100 days. But the danger would not have passed since the incidence will be around 2500. In Cuba, according to official reports, 23% of the total population have been vaccinated, until August 1, 2021, but in the following 140 days, (until the end of the year 2021), 47% of the total population would have been immunized. Which is still insufficient to achieve the necessary immunity against the delta variant, which currently circulates in 13 provinces of the country. For this reason, it is necessary to achieve greater vaccination coverage, proposing that by the end of the year 80% of the population vaccinated be reached. Economic difficulties and shortages force greater mobility of people, reducing the possibilities of establishing total isolation from the population.

**FIG. 7.**
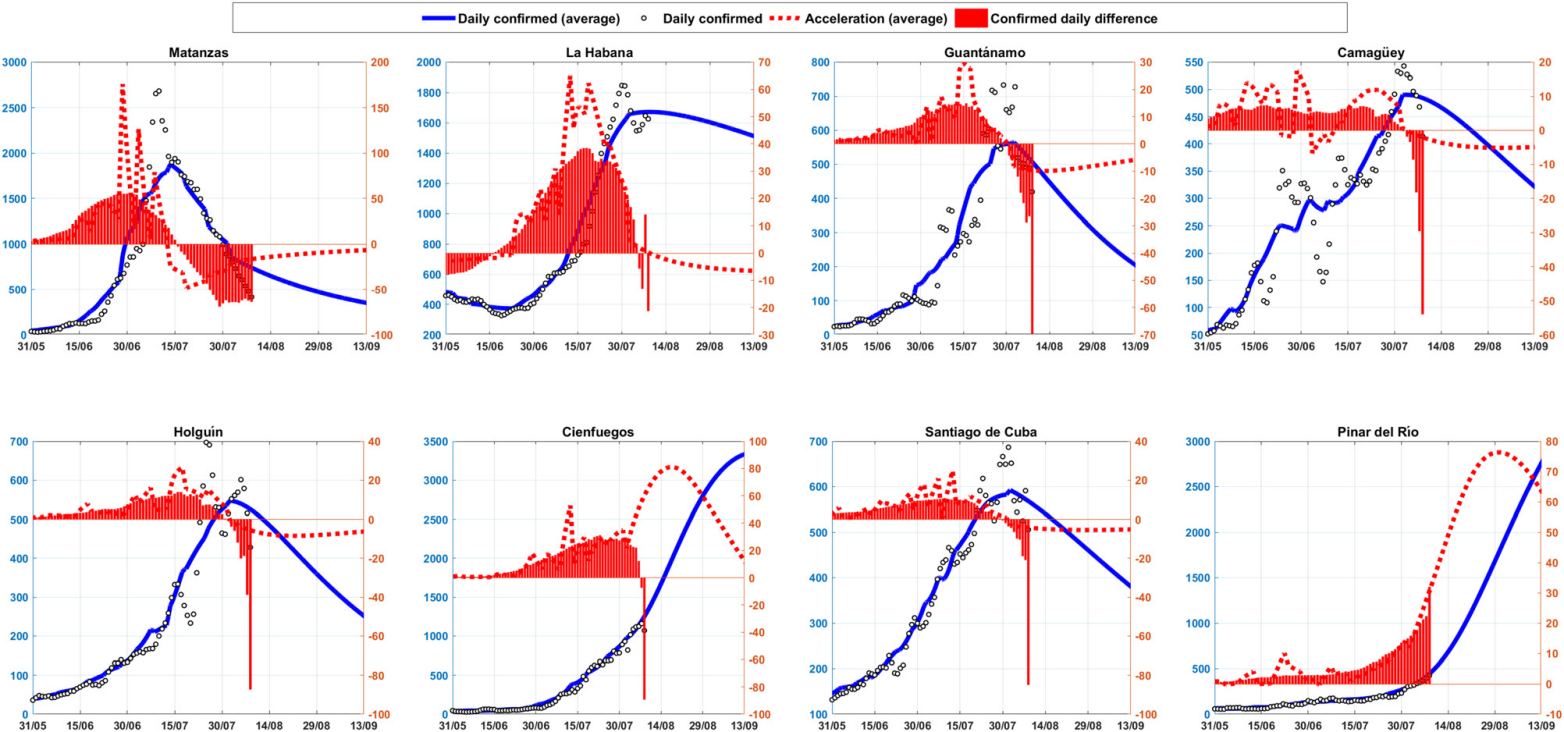
The daily acceleration of the cases for the provinces Matanzas, La Habana, Guantanamo, Camagüey, Holguín, Cienfuegos, Santiago de Cuba, and Pinar del Río.

**FIG. 8.**
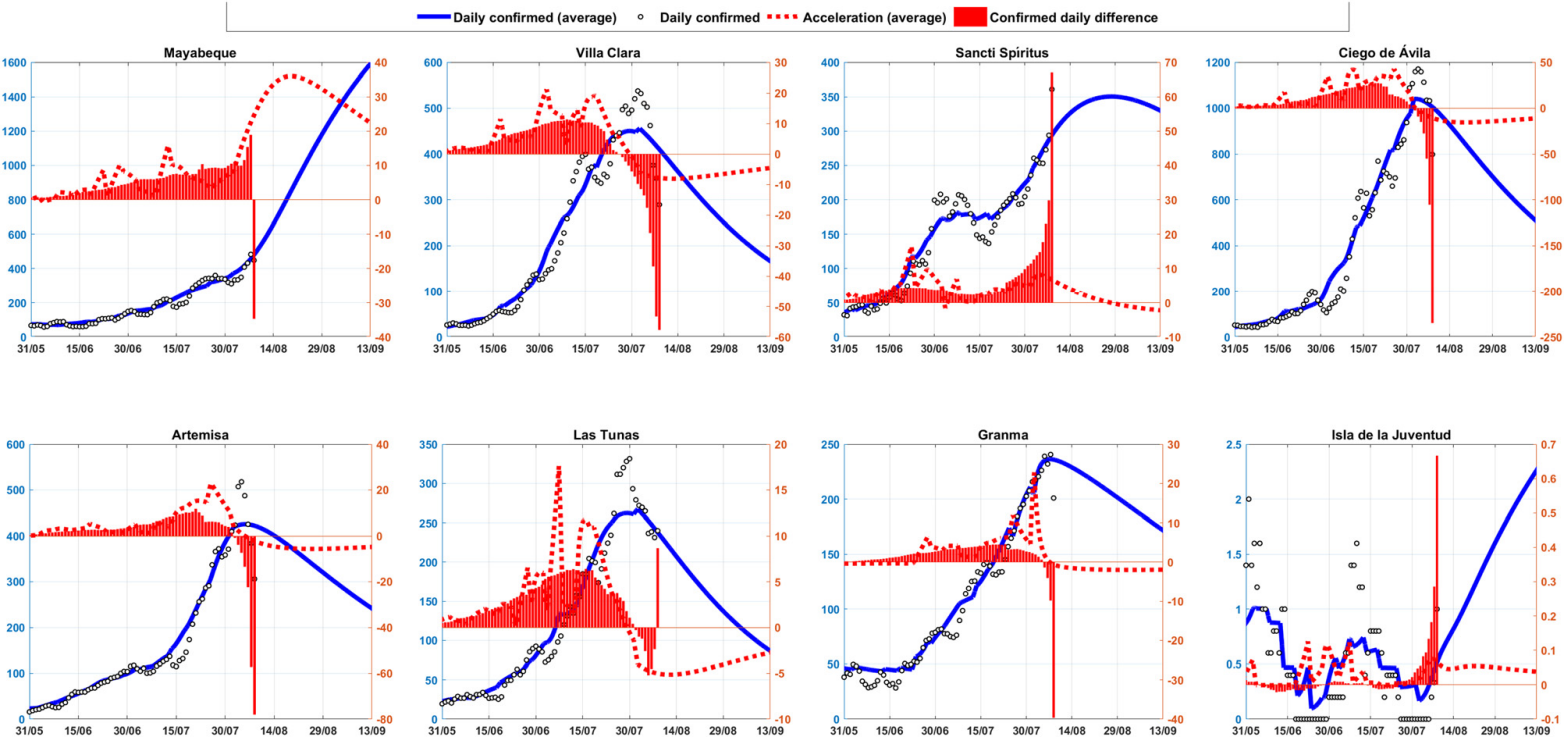
The daily acceleration of the cases for the provinces Mayabeque, Villa Clara, Santi Spiritus, Ciego de Avila, Artemisa, Las Tunas, Granma, and Isla de la Juventud.

In Figs. 7 and 8, the rate of acceleration of confirmed cases for the Cubans provinces is shown through the red lines and bars. The dashed red line denotes the forecast and bars denote the daily report. This is the rate at which the number of new cases in the Cuban provinces is accelerating or slowing down, which detects whether or not the red dashed lines are above the red horizontal axis respectively. Blue shows the prognostics of daily confirmed cases.

The epidemiological situation in Cuba at this moment (8 august) is very complicated. It is observed that the provinces of Pinar del Río, Cienfuegos, Mayabeque, Ciego and Santiago, show an acceleration in the growth of cases, while in Matanzas, Havana and Guantánamo the appearance of new cases is slowing down, but with many confirmed cases. With less incidence, but the processes also accelerate in Artemisa, Sancti Spíritus, Camagüey, and Granma.

In a more in-depth analysis, the behavior of the reproductive number *R*_0_ associated with the system (2) is studied from March 2021 to October 2021. The expression of *R*_0_ is deduced in Appendix A. By taking into account the parameters that are obtained by fitting the curves to the data, the variation of the reproductive number can be appreciated. Before starting the vaccination period in Cuba, the reproductive number reached 4.5, indicating a high transmission of the virus. By the beginning of May, *R*_0_ got to be below 1. But at the end of the same month, the delta variant began to spread throughout the country, which was reflected in the reproductive number that again reached values above 2. This indicates a high transmission and a deterioration in the control of the epidemic. Even on this date, the disease-free equilibrium point is considered unstable, only until October; the parameters will be achieved to guarantee its stability.

## V. COMPARISON OF THE BEHAVIOR OF THE PANDEMIC BETWEEN CUBA AND OTHER COUNTRIES

This section shows a comparison of the behavior of the pandemic in 2021 among various countries. The objective is to compare the trajectory that the pandemic has followed in countries of different regions of the world and Cuba. For the study, data from Chile (18.95 million), Israel (9.053 million), United Kingdom (66.65 million), Serbia (6.945 million), and United Arab Emirates (9.771 million) were taken. The data are reported on the website Datosmacro.com.^29^

**FIG. 9.**
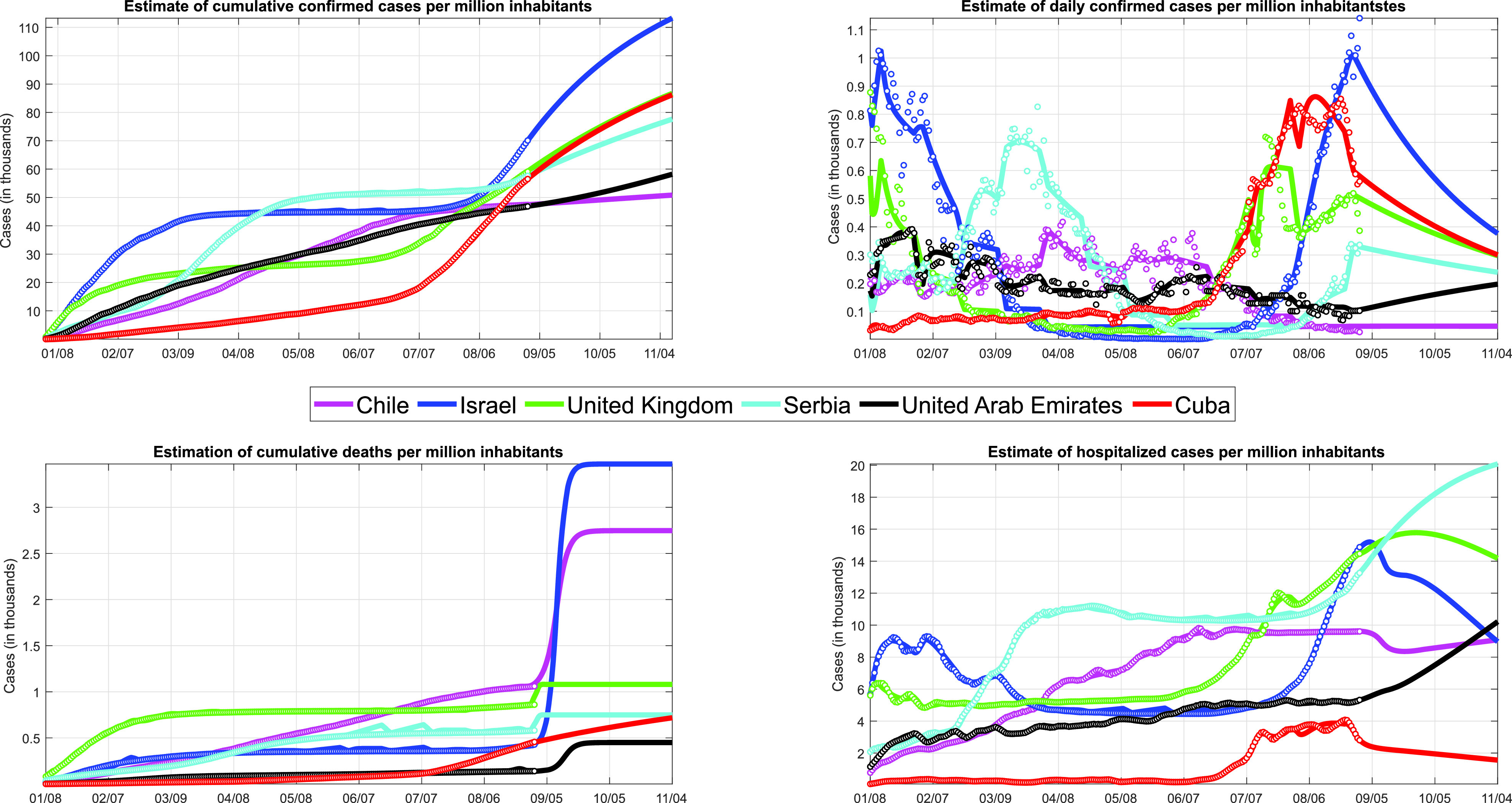
Comparison of the behavior of the pandemic between Chile, Israel, United Kingdom, Serbia, United Arab Emirates, and Cuba from January 8, 2021, to August 30, 2021.

Figure 9 shows a comparison between the different countries, taking the data presented in 29 and the prediction given by the model using system (2). To illustrate, four different curves were used that represent the number of confirmed cases, the number of confirmed daily cases, the number of deaths, and the number of hospitalized cases. It is observed that during the beginning of the year Cuba managed to maintain low rates of virus infestation and a low fatality rate, compared to countries with less population such as Israel, Serbia, and the United Arab Emirates. These situations have deteriorated in the summer, with the entry of the delta strain and the weakening of distancing measures and now Cuba is among the countries with the highest incidence, with more than 6 thousand cases per day, but maintaining a low cumulative fatality than most of the countries mentioned in the comparison. It is important to note that the countries used in the comparison have a high vaccination rate of between 40 and 75% of the fully vaccinated population.

## VI. CONCLUSIONS

The mathematical models presented here are an effective generalization of classic SEIR models to the case of multiple populations and the vaccination process. As we have seen in the results, the model has been able to reproduce and predict with great precision the behavior of the COVID-19 virus in Cuba during the first year. It was efficiently illustrated how the interaction between the populations changed the course of the pandemic for each of the regions of Cuba. On the other hand, an exhaustive analysis of the vaccination process is presented, using the official vaccination schedule presented by the Cuban Ministry of Health. The model shows control of the pandemic taking into account the project proposed by the Cuban Ministry of Health and how the vaccine becomes essential for the protection of the population. The results have enabled MINSAP to anticipate the needs for medicines, medical supplies, and health personnel needed to deal with this contingency. The national pharmaceutical industry has taken them into account to provide the health system with antivirals, breathing equipment, and other supplies necessary to face the pandemic. The provincial governments have relied on these predictions to protect the population and avoid further impacts. The model has several applications and adaptations, among which the following stand out: the study of the influence of the vaccine in the control of COVID-19, effectiveness of the vaccine based on the protection ratio, and the interaction among different mutations of the virus. The same model can easily be used for different studies of the spread of epidemics and the interaction among different age groups, races, or communities.

## Data Availability

The data that support the findings of this study are openly available in COVID19 Cuba Data at https://covid19cubadata.github.io/#cuba, Ref. 19 and Datosmacro.com at https://datosmacro.expansion.com/otros/coronavirus-vacuna.

## APPENDIX A: DEDUCTION OF THE BASIC REPRODUCTION NUMBER

It is relevant to determine the threshold value of the disease or the basic reproduction number, denoted by *R*_0_. It can be used not only to explore the stability of break-even points but also to report whether the disease will disappear or persist in a population.

For this deduction, the value of *R*_0_ for system (2) is determined. Using a change of variable for each of the subpopulations of the system,

$$s=\frac{S}{N},\;e=\frac{E}{N},\;i=\frac{I}{N},\;q=\frac{Q}{N},\;r=\frac{R}{N},\;d=\frac{D}{N},\;p=\frac{P}{N}.$$

System (2) becomes

$$\begin{array}{cc} & \frac{ds}{dt}=-\beta si-\alpha s-\eta \nu s,\\ & \frac{de}{dt}=\beta si-\gamma e+\beta (1-\eta )(1-\alpha )pi,\\ & \frac{di}{dt}=\gamma e-\delta i,\\ & \frac{dq}{dt}=\delta i-\lambda q-\kappa q,\\ & \frac{dp}{dt}=(\alpha +\eta \nu )s-\beta (1-\eta )(1-\alpha )pi,\\ & \frac{dr}{dt}=\lambda q,\\ & \frac{dd}{dt}=\kappa q. \end{array}$$
(A1)

Notice the last two equations of the system (A1) are uncoupled. Therefore, for the analysis only the first 5 equations of the system (A1) are taken into account. Two equilibrium points for the system in the form P=(s,e,i,q,p) are obtained. The point $P_{0}=(0,0,0,0,1)$ a disease-free equilibrium point and $P_{1}=(s_{1},e_{1},i_{1},q_{1},p_{1})$ as an endemic equilibrium point. Second equilibrium emerges as a non-simple form so that it is stated only by $P_{1}$. Hence, the analysis was conducted only for disease-free equilibrium.

The Jacobian matrix *J* for the system of s,e,i,q,p at $P_{0}$ is

$$J(P_{0})=\left(\begin{array}{ccccc} -(\alpha +\eta v) & 0 & 0 & 0 & 0\\ 0 & -\gamma & \beta (1-\eta )(1-\alpha ) & 0 & 0\\ 0 & \gamma & -\delta & 0 & 0\\ 0 & 0 & \delta & -(\lambda +\kappa ) & 0\\ \left(\alpha +\eta v\right) & 0 & -\beta (1-\eta )(1-\alpha ) & 0 & 0 \end{array}\right).$$

The corresponding eigenvalues for *J* are

$$\begin{array}{cc} & h_{1}=0,\\ & h_{2}=-\left(\alpha +\eta v\right),\\ & h_{3}=-\left(\lambda +\kappa \right),\\ & h_{4,5}=-\frac{1}{2}(\gamma +\delta )\pm \frac{1}{2}\sqrt{{\left(\gamma -\delta \right)}^{2}+4\gamma \beta (1-\eta )(1-\alpha )}. \end{array}$$
(A2)

Imposing the condition that $h_{4,5}<0$, it is obtained that

−δ+β(1−η)(1−α)<0,

and finally, the following relationship is derived:

$$\frac{\beta (1-\eta )(1-\alpha )}{\delta }<1.$$
(A3)

Under the assumption that $\frac{\beta (1-\eta )(1-\alpha )}{\delta }>1$ then the disease-free equilibrium point *P*_0_ will be unstable. In the other words, the endemic equilibrium *P*_1_ will exist. From (A3), the basic reproduction number is derived,

$$R_{0}=\frac{\beta (1-\eta )(1-\alpha )}{\delta }.$$
(A4)

## APPENDIX B: DEDUCTION OF THE ACCELERATION FORMULA

In this section, the acceleration formula (3) From system (2), we get that

$$\begin{array}{c} \frac{dQ}{dt}+\frac{dR}{dt}+\frac{dD}{dt}=\delta I+\lambda Q-\kappa Q+\lambda Q+\kappa Q,\\ \frac{d}{dt}(Q+R+D)=\delta I. \end{array}$$
(B1)

Taking the derivative in both sides, it is obtained

$$\begin{array}{c} \frac{d^{2}}{dt^{2}}(Q+R+D)=\delta \frac{dI}{dt},\\ \frac{d^{2}}{dt^{2}}(Q+R+D)=\delta \left(\gamma E-\delta I\right),\\ \frac{d^{2}}{dt^{2}}(Q+R+D)=\delta \gamma E-\delta^{2}I. \end{array}$$
(B2)
